# The critical role of interleukin-6 in protection against neurotropic flavivirus infection

**DOI:** 10.3389/fcimb.2023.1275823

**Published:** 2023-11-16

**Authors:** Tabassum T. Auroni, Komal Arora, Janhavi P. Natekar, Heather Pathak, Amany Elsharkawy, Mukesh Kumar

**Affiliations:** Department of Biology, College of Arts and Sciences, Georgia State University, Atlanta, GA, United States

**Keywords:** West Nile virus, Japanese encephalitis virus, flavivirus, interleukin-6 (IL-6), host pathogen interaction, neuronal cells, mouse models, encephalitis

## Abstract

West Nile virus (WNV) and Japanese encephalitis virus (JEV) are emerging mosquito-borne flaviviruses causing encephalitis globally. No specific drug or therapy exists to treat flavivirus-induced neurological diseases. The lack of specific therapeutics underscores an urgent need to determine the function of important host factors involved in flavivirus replication and disease progression. Interleukin-6 (IL-6) upregulation has been observed during viral infections in both mice and humans, implying that it may influence the disease outcome significantly. Herein, we investigated the function of IL-6 in the pathogenesis of neurotropic flavivirus infections. First, we examined the role of IL-6 in flavivirus-infected human neuroblastoma cells, SK-N-SH, and found that IL-6 neutralization increased the WNV or JEV replication and inhibited the expression of key cytokines. We further evaluated the role of IL-6 by infecting primary mouse cells derived from IL-6 knockout (IL-6^−/−^) mice and wild-type (WT) mice with WNV or JEV. The results exhibited increased virus yields in the cells lacking the IL-6 gene. Next, our *in vivo* approach revealed that IL-6^−/−^ mice had significantly higher morbidity and mortality after subcutaneous infection with the pathogenic WNV NY99 or JEV Nakayama strain compared to WT mice. The non-pathogenic WNV Eg101 strain did not cause mortality in WT mice but resulted in 60% mortality in IL-6^−/−^ mice, indicating that IL-6 is required for the survival of mice after the peripheral inoculation of WNV or JEV. We also observed significantly higher viremia and brain viral load in IL-6^−/−^ mice than in WT mice. Subsequently, we explored innate immune responses in WT and IL-6^−/−^ mice after WNV NY99 infection. Our data demonstrated that the IL-6^−/−^ mice had reduced levels of key cytokines in the serum during early infection but elevated levels of proinflammatory cytokines in the brain later, along with suppressed anti-inflammatory cytokines. In addition, mRNA expression of IFN-α and IFN-β was significantly lower in the infected IL-6^−/−^ mice. In conclusion, these data suggest that the lack of IL-6 exacerbates WNV or JEV infection *in vitro* and *in vivo* by causing an increase in virus replication and dysregulating host immune response.

## Introduction

West Nile virus (WNV) and Japanese encephalitis virus (JEV) are emerging mosquito-borne flaviviruses that have caused global epidemics of viral encephalitis (Pierson and Diamond, 2020). Since its arrival in New York in 1999 (Nash et al., 2001), WNV, an NIAID Category B Priority Pathogen, has become the main cause of arboviral encephalitis in the United States, resulting in over 7 million infections, 28,000 neuroinvasive disease cases, 20,000 hospitalizations, and 2,600 deaths between 1999 and 2022 (Shocket et al., 2020; Virus, 2022). Similarly, JEV is the leading cause of virus-induced encephalitis in Asia, responsible for approximately 68,000 cases annually and an estimated 20,000 fatalities, with nearly half of the survivors experiencing long-term neurologic sequelae (Moore, 2021). Aside from encephalitis, both viruses can cause severe neurological diseases such as meningitis, paralysis, and death (Misra and Kalita, 2010; Peng and Wang, 2019). These viruses predominantly affect gray matter and prefer targeting specific regions of the nervous system, including the basal ganglia, cerebellum, and spinal cord (Clarke et al., 2014). Neurons are their prime target while replicating in the brain (Sips et al., 2012). The subsequent pathology is marked by neuronal loss, glial cell activation, blood-brain barrier disruption, and leukocyte infiltration to the parenchyma and perivascular space (Mathur et al., 1992; Zhou et al., 2003; Van Marle et al., 2007; Das et al., 2008; Chen et al., 2010; Stewart et al., 2011; Roe et al., 2012; Mustafá et al., 2019; Peng and Wang, 2019). Both viruses continue to spread and are causing human diseases in new parts of the world (Petersen and Marfin, 2005). However, no effective therapies are available for treating individuals infected with WNV or JEV.

Interleukin-6 (IL-6) is a key cytokine that regulates various aspects of the host immune response. It has a broad range of effects on inflammation, autoimmunity, and acute phase response (Banks et al., 1995; Ishihara and Hirano, 2002; Tanaka et al., 2014; Narazaki and Kishimoto, 2018). Moreover, IL-6 is upregulated during viral and bacterial infections in mice and humans (Soda et al., 2003; Velazquez-Salinas et al., 2019), implying that it may also play an important role in disease pathogenesis. Consistent with others’ studies, we have previously demonstrated that WNV infection induces a dramatic upregulation of IL-6 in mouse brains and primary mouse cells, including mouse embryonic fibroblasts (MEFs), macrophages, dendritic cells, astrocytes, and neurons (Kumar et al., 2010; Natekar et al., 2020). Interestingly, several investigations have demonstrated the protective role of IL-6 during viral infections. For example, the neutralization of endogenous IL-6 increased the virus burden following hepatitis B infection *in vitro* (Kuo et al., 2009). Furthermore, IL-6-deficient mice were used to reveal the importance of IL-6 for the survival of mice infected with the influenza virus and herpes simplex virus (LeBlanc et al., 1999; Lauder et al., 2013; Yang et al., 2017). Another study showed that blocking IL-6 or IL-6R (IL-6 receptor) by monoclonal antibodies during lymphocyte choriomeningitis virus (LCMV) infection restricted virus clearance in mice (Harker et al., 2011). Similarly, disruption of the IL-6 gene in mice impaired the immune response after infection with the vaccinia virus (Kopf et al., 1994). However, our knowledge of the function of IL-6 during the progression of a neurotropic flavivirus infection is lacking.

In the present study, we show the critical role of IL-6 in the neurotropic infections induced by WNV or JEV in human neuronal cells, primary murine cells, and mice. Our data revealed that human neuronal cells, primary murine neuronal cultures, mouse embryonic fibroblasts (MEFs), and bone marrow-derived macrophages (BMDMs) lacking IL-6 produced higher virus titers compared to the corresponding cells derived from wild-type (WT) mice upon infection with WNV or JEV. The IL-6^−/−^ mice exhibited increased morbidity and mortality than WT mice when infected with either lethal or non-lethal strains of WNV or JEV. Higher viral loads were observed in the serum and brains of IL-6^−/−^ mice at different time points of the infection. Additionally, reduced antiviral interferon response and cytokine production were observed in the absence of IL-6. Collectively, these results provide the first evidence of the important role of IL-6 in limiting the severity of WNV and JEV infections.

## Materials and methods

### Flavivirus infection and IL-6 neutralization in human neuroblastoma cells

A transformed human neuroblastoma cell line, SK-N-SH, was purchased from the American Tissue Culture Collection and propagated as described previously (Kumar et al., 2010). Cells below passage 10 were used for all experiments. SK-N-SH cells seeded in 12-well plates (1.2 × 10^5^ cells/well) were infected with WNV NY99 or JEV Nakayama strain at the multiplicity of infection (MOI) of 0.1 as described previously (Kumar et al., 2010). Briefly, the cells were incubated with virus for 1 hour at 37°C. After incubation, the cells were replenished with fresh media or media containing neutralizing monoclonal antibodies against IL-6 (Catalog # MAB2061, R&D Systems). The concentrations of anti-IL-6 antibodies employed in this study were 5, 10, and 20 μg/mL. Supernatant and cell lysates were collected at 12, 24, and 48 hours (Murphy et al., 2008). The viral load in the supernatant was measured by plaque formation assay using Vero cells as described previously (Natekar et al., 2022).

### Enzyme-linked immune sorbent assay (ELISA)

IL-6 neutralization was confirmed by measuring the protein levels of IL-6 in the WNV-infected SK-N-SH cell culture supernatants using the Quantakine Human IL-6 ELISA kit (R&D Systems) (Kumar et al., 2010). The tests were conducted following the guidelines provided by the manufacturer, and the plates were analyzed using a Victor 3 microtiter reader as described previously (Kumar et al., 2010).

### Cell viability assay

SK-N-SH cells were seeded in 96-well plates (2 × 10^4^ cells/well) and treated with 5, 10, and 20 μg/mL anti-IL-6 antibodies for 48 hours. Then, cell viability was assessed using CellTiter 96 AQueous One Solution Cell Proliferation Assay (Promega) as described previously (Rothan et al., 2019).

### Quantitative real-time reverse transcriptase-PCR analysis

Total RNA was isolated from WNV-infected SK-N-SH cell lysates using a Qiagen RNeasy Mini kit (Qiagen, Germantown, MD, USA) and utilized to generate cDNAs using an iScript™ cDNA synthesis kit (Bio-Rad, Catalog #1708891). Quantitative RT-PCR (qRT-PCR) was conducted using the produced cDNAs utilizing SsoAdvanced™ Universal SYBR^®^ Green Supermix (Biorad, Catalog # 1725271) to determine the expression levels of key cytokines (Stone et al., 2021). The fold change in WNV-infected cells compared to mock-infected cells was calculated after normalizing to the housekeeping GAPDH gene (Natekar et al., 2020).

### Animals

C57BL/6J (WT) mice and IL-6^−/−^ mice (002650) were acquired from the Jackson Laboratory (Bar Harbor, ME, USA). These mice were bred and genotyped in the animal facility located at Georgia State University. All WNV and JEV infection experiments were conducted in an animal biosafety level-3 (ABSL-3) laboratory. This study was performed following the National Institutes of Health and the Institutional Animal Care and Use Committee (IACUC) guidelines. The protocol was validated by the Georgia State University IACUC (protocol number A21067).

### WNV and JEV infection of primary mouse cells

One-day-old pups from established colonies of WT and IL-6^−/−^ mice were used to isolate MEFs and mouse cortical neurons as previously described (Durkin et al., 2013; Azouz et al., 2019). MEFs were cultured in DMEM media containing 10% FBS and 1% penicillin/streptomycin antibiotic. Neurons were seeded on poly-D-lysine-coated plates and maintained in serum-free neurobasal A media supplemented with B27 (Gibco) for one week before infection. For isolating BMDM, eight-week-old WT and IL-6^−/−^ mice were euthanized, and bone marrow cells were generated from the hind limbs as previously described (Kumar et al., 2013). The cultures were maintained in DMEM media supplemented with 10% FBS and 40 ng/mL macrophage colony-stimulating factor for one week before flavivirus infection.

MEFs, BMDMs, and neuronal cultures were infected with JEV Nakayama, WNV NY99, or WNV Eg101 at the MOI of 0.1 for one hour or inoculated with PBS (mock). Supernatants and cell lysates were collected at 12, 24, 48, and 72 hours after infection. Virus titers were then measured in the culture supernatants by plaque assay as previously described (Verma et al., 2009).

### IL-6 deletion efficiency in primary mouse cells

Total RNA was extracted from MEFs and BMDMs cell pellets using a Qiagen RNeasy Mini kit (Qiagen) and used to generate cDNAs utilizing an iScriptTM cDNA synthesis kit (Bio-Rad). qRT-PCR was performed using the cDNAs to quantify the expression levels of IL-6 as previously described (Stone et al., 2021). The fold change in IL-6 expression in IL-6^−/−^ cells, relative to WT cells, was calculated after normalizing to the housekeeping GAPDH gene (Natekar et al., 2020).

For the gel electrophoresis, a 2% agarose gel was prepared using 1X TAE (Tris-Acetate-EDTA) buffer. Once the gel solidified, a mixture of 2 µl of 6X SYBR Green loading dye and 10 µl of the qRT-PCR product were loaded into each lane. Additionally, a 10 µl sample of a 100-bp ladder was loaded into one lane as a size reference. The gel was initially electrophorized at 100 V for 15 minutes and then at 110 V for 45 minutes. The gel was visualized using BioDoc-It™ 220 imaging system (UVP, Upland, CA, USA).

### Animal infection experiments and plaque assay

Eight-week-old WT and IL-6^−/−^ mice were transferred to the ABSL-3 facility and acclimated to the local surroundings before initiating experiments. For survival studies, WT and IL-6^−/−^ mice were inoculated with PBS (mock) or 100 PFU of WNV NY99 strain or 1,000 PFU of WNV Eg101 strain or 1,000 PFU of JEV Nakayama strain via the subcutaneous route. On days 2, 3, 4, and 6 after inoculation, 100 μL of blood was drawn from the tail veins to determine viremia and serum protein levels of cytokines and chemokines. These animals were monitored daily for clinical signs until day 21. A clinical scoring sheet was utilized to evaluate their appearance (coat condition, posture, grooming), signs of neurological disease (lethargy, ataxia, paralysis), and behavior (active, subdued when stimulated, unresponsiveness). Mice displaying severe symptoms, such as tremors and paralysis, were euthanized by CO_2_ inhalation followed by cervical dislocation. In separate experiments, WT and IL-6^−/−^ mice were subcutaneously inoculated with PBS, WNV NY99 (100 PFU), WNV Eg101 (1,000 PFU), or JEV Nakayama (1,000 PFU). On days 3, 6, and 8 after infection, the mice were anesthetized with isoflurane, and blood was collected through heart puncture. The mice were then perfused with PBS, and their brains were harvested and flash-frozen in 2-methyl butane, followed by tissue homogenization. Serum and brain homogenates were analyzed by performing plaque assays as previously described (Verma et al., 2009) to quantify WNV and JEV titers. Viral loads were expressed as PFU per milliliter of serum and PFU per gram of brain tissue.

### Multiplex immunoassay

The protein concentrations of inflammatory cytokines and chemokines in the mouse serum and brain homogenates were measured using a multiplex immunoassay kit (MILLIPLEX MAP Mouse Cytokine/Chemokine Kit, Millipore) (Kumar et al., 2012).

### Type I interferon expression in mouse brains

Total RNA isolation from the brain homogenates was performed using a Qiagen RNeasy Mini kit (Qiagen), adhering to the manufacturer’s instructions. cDNA was synthesized using an iScript™ cDNA synthesis kit (Biorad) and was employed for qRT-PCR to determine the expression levels of IFN-α and IFN-β (Stone et al., 2021). The fold change in their expressions within the infected tissue was calculated against mock-infected tissue after normalization to the GAPDH gene (Natekar et al., 2020).

### Statistical analysis

GraphPad Prism 7.0 was used to perform a two-way analysis of variance (ANOVA) to measure *p* values for WNV and JEV titers in cell cultures, IL-6 protein levels in cell culture supernatants, the fold changes of cytokine expressions, IL-6 gene deletion efficiency test in primary mouse cells, type I IFN expression in mouse brain, and the quantification of the proteins in mouse serum. The Mann–Whitney test was used to calculate the *p* values for the viability test of antibody-treated SK-N-SH cells, plaque assay titers in mouse serum and brain, and the protein levels of various cytokines and chemokines in brains. Survival curves were compared using a Kaplan–Meier log-rank test. Unpaired Student’s *t*-test was applied to measure *p* for the clinical scores. *p* values < 0.05 were used to indicate a statistically significant difference.

## Results

### Neutralization of IL-6 in flavivirus-infected human neuroblastoma cells causes an increase in viral load and a decrease in proinflammatory cytokine expression levels

**Figure 1:**
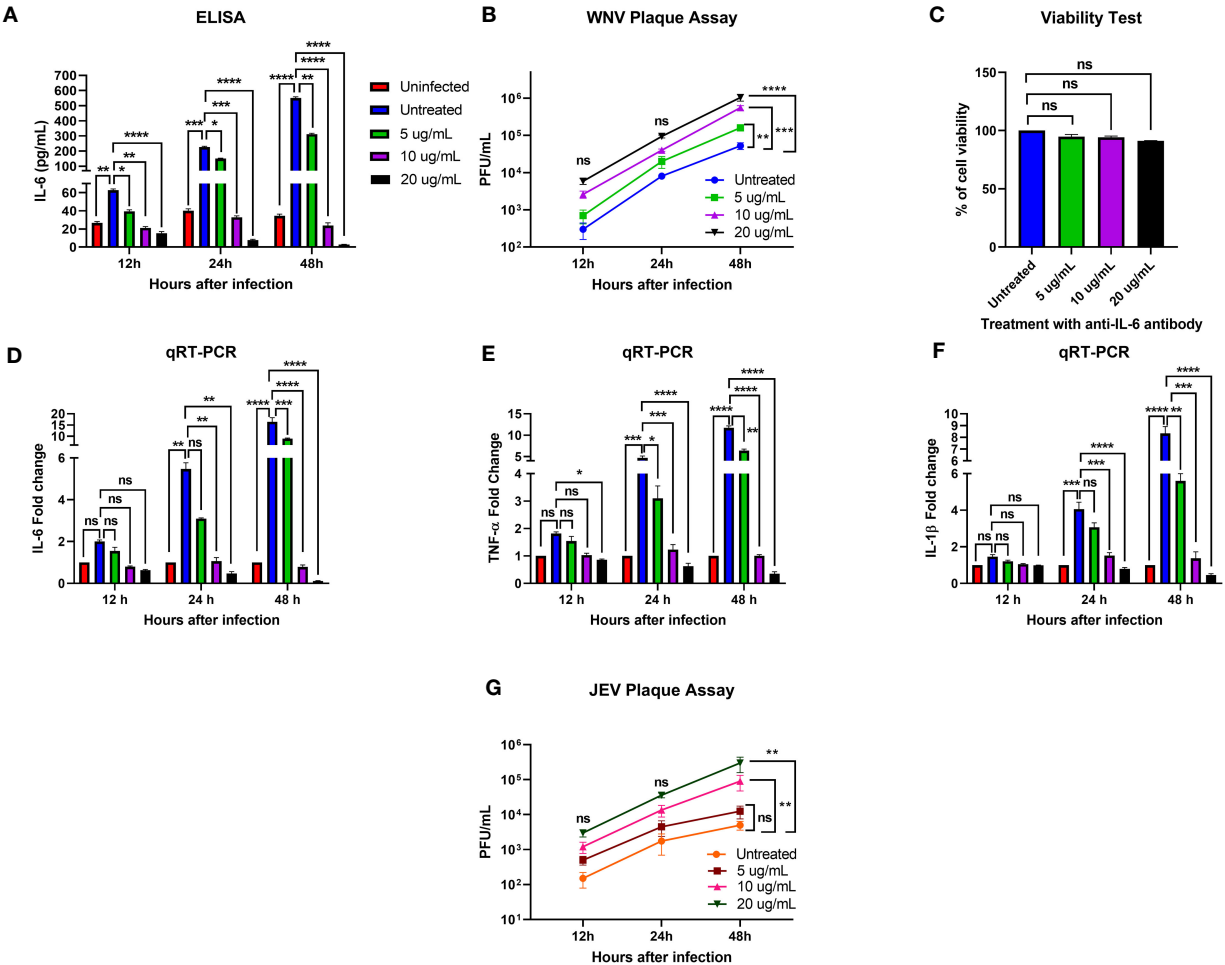
Effect of IL-6 neutralization in flavivirus-infected human neuronal cells. The human neuroblastoma cell line, SK-N-SH, was infected with WNV NY99 or JEV Nakayama strain (as described in Materials and Methods), followed by treatment with 5, 10, and 20 ug/mL of monoclonal antibody against IL-6. The supernatant and cell lysates were collected at different time points. **(A)** IL-6 protein levels were measured in the cell culture supernatant by ELISA. The data expressed are the mean concentration (pg/mL) ± SD of IL-6 in the supernatant, representing two separate experiments. **(B, G)** Viral loads in the cell culture supernatants were quantified by plaque assay. Results from two independent studies in duplicate are presented as PFU/mL ± SD. **(C)** Cell viability of SK-N-SH cells was assessed by conducting a cell proliferation assay at 48 hours after antibody treatment. Data are presented as mean ± SD for two independent experiments performed in triplicate. **(D–F)** cDNA templates from mock, WNV-infected, and anti-IL-6 antibody-treated WNV-infected SK-N-SH cells were used to evaluate the fold-change of IL-6, TNF-α, and IL-1β at different time points using qRT-PCR. Changes in the levels of these cytokines were normalized to the housekeeping gene, GAPDH, and the fold change in infected cells as compared to mock-infected controls was calculated. Data represent mean ± SD of three independent experiments conducted in duplicate. Statistical significance is presented as *p < 0.05, **p < 0.01, ***p < 0.001, ****p < 0.0001.

To determine the role of IL-6 in human neuronal cells during flavivirus infection, we infected human neuroblastoma cells, SK-N-SH, with WNV NY99 or JEV Nakayama strain at the MOI of 0.1 and treated these infected cells with various concentrations of anti-IL-6 antibody. To verify the neutralization of IL-6 in the antibody-treated samples, we performed an ELISA to measure the IL-6 levels in culture supernatants obtained from WNV infection. We observed very low levels of IL-6 in mock-infected cells. There was a significant upregulation of IL-6 levels in the infected cells without antibody treatment at all time timepoints. As expected, treatment with anti-IL-6 antibody decreased the IL-6 concentration significantly in the infected cells. Our results showed that the antibody effectively blocked IL-6 in a time- and dose-dependent manner, evidenced by the lowest concentration of IL-6 (2.8 pg/mL) measured after 48 hours of treatment with 20 ug/mL antibody (**Figure 1A**).

To examine the effect of IL-6 neutralization on the susceptibility of SK-N-SH cells to WNV or JEV infection, we analyzed virus titers in the culture supernatants of infected and antibody-treated cells collected at 12, 24, and 48 hours after infection. Plaque assay data demonstrated that all samples had gradually increased virus yields from 12 to 48 hours. When comparing the virus titers between infected samples with and without antibody treatment, WNV and JEV loads were higher in the antibody-treated samples at each time point. However, the increase in viral loads reached statistical significance in a dose-dependent manner only at 48 hours (**Figures 1B, G**). These data suggest that blocking IL-6 in SK-N-SH cells enhances WNV and JEV replication.

We also assessed the viability of antibody-treated SK-N-SH cells to determine the possible cytotoxicity effect of 5, 10, and 20 ug/mL anti-IL-6 antibody on the cells after 48 hours of treatment. A cell proliferation assay revealed that the antibody doses employed in this experiment did not cause significant cell death (**Figure 1C**).

We next investigated the effect of IL-6 neutralization on the expression of key proinflammatory cytokines, TNF-α and IL-1β, in mock- and WNV-infected SK-N-SH cells by performing a qRT-PCR using RNA extracted from the cell lysates. TNF-α and IL-1β are known to initiate innate immune response, and mediate the activation, recruitment, and adherence of circulating macrophages and neutrophils to the infection site (Dinarello, 2000). We observed a significant upregulation of IL-6, TNF-α, and IL-1β in the infected cells compared to mock-infected cells at 24 and 48 hours. However, despite higher viral load, treatment with 10 and 20 ug/mL concentrations of anti-IL-6 antibody led to a significant downregulation of these cytokines in the infected cells after 24 and 48 hours (**Figures 1D–F**). The data from this experiment indicate that IL-6 neutralization inhibited the expression of critical cytokines, TNF-α and IL-1β, in the WNV-infected cells.

### IL-6 restricts WNV and JEV replication in primary mouse cells

**Figure 2:**
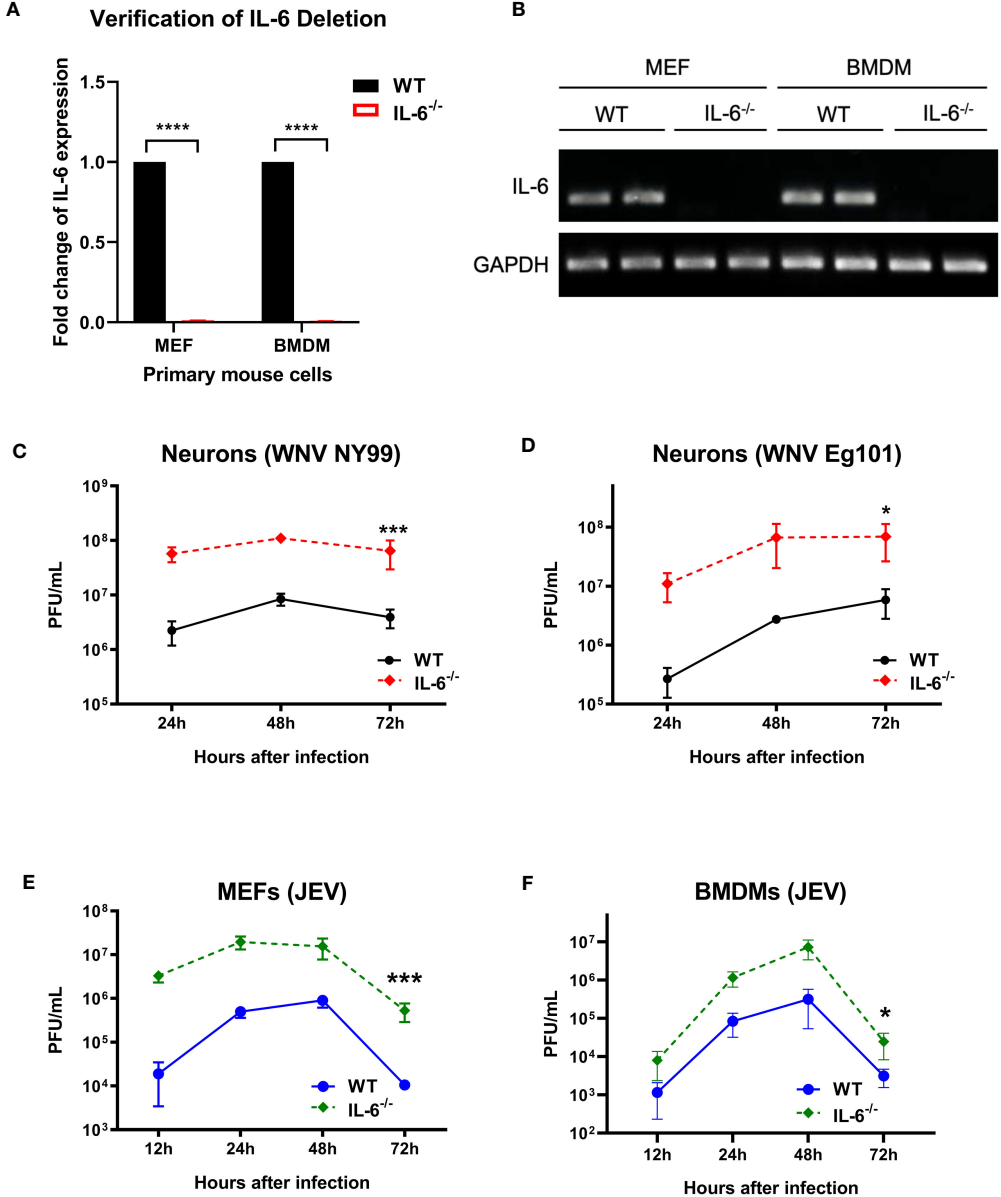
Flavivirus titers in the infected primary mouse cells isolated from WT and IL-6^−/−^ mice. **(A, B)** qRT-PCR and gel electrophoresis were performed to confirm the deletion of IL-6 gene in primary cells derived from IL-6**^−/−^** mice. **(C)** WNV NY99, **(D)** WNV Eg101, or **(E, F)** JEV Nakayama were used to infect neuronal cultures, MEFs, and BMDMs (as described in Materials and Methods), and viral titers in the cell culture supernatants were determined at various time points by plaque assay. Results from at least three separate studies carried out in duplicate are presented as PFU/mL ± SD. *p < 0.05, ***p < 0.001, ****p < 0.0001.

To further define the role of IL-6 during flavivirus infection, we planned an *in vitro* experiment using primary murine cells. We performed a comprehensive analysis of virus growth in cortical neurons, MEFs, and BMDMs isolated from WT and IL-6^−/−^ mice. The absence of the IL-6 gene in the IL-6^−/−^ cells was first verified by qRT-PCR and gel electrophoresis. No expression of IL-6 was detected in the cells derived from the IL-6^−/−^ mice (**Figures 2A, B**). These primary cells were then infected with WNV NY99, WNV Eg101, or JEV Nakayama strain at the MOI of 0.1, and the supernatants were harvested at various time points after infection (12, 24, 48, and 72 hours). Virus titers were then quantified in the cell supernatants using plaque assays. We observed that neuronal cultures derived from IL-6^−/−^ mice exhibited significantly higher virus titers upon WNV NY99 or WNV Eg101 infection than those from WT mice (**Figures 2C, D**). Furthermore, MEFs and BMDMs isolated from IL-6^−/−^ mice produced significantly increased virus yields after infection with JEV compared to cells derived from WT mice (**Figures 2E, F**). These experiments show that the knockout of the IL-6 gene increases the replication of lethal or non-lethal strains of WNV or JEV in primary mouse cells derived from both peripheral tissues and the CNS, indicating the protective role of IL-6 in both areas.

### IL-6 limits WNV and JEV pathogenesis in mice following peripheral infection

**Figure 3:**
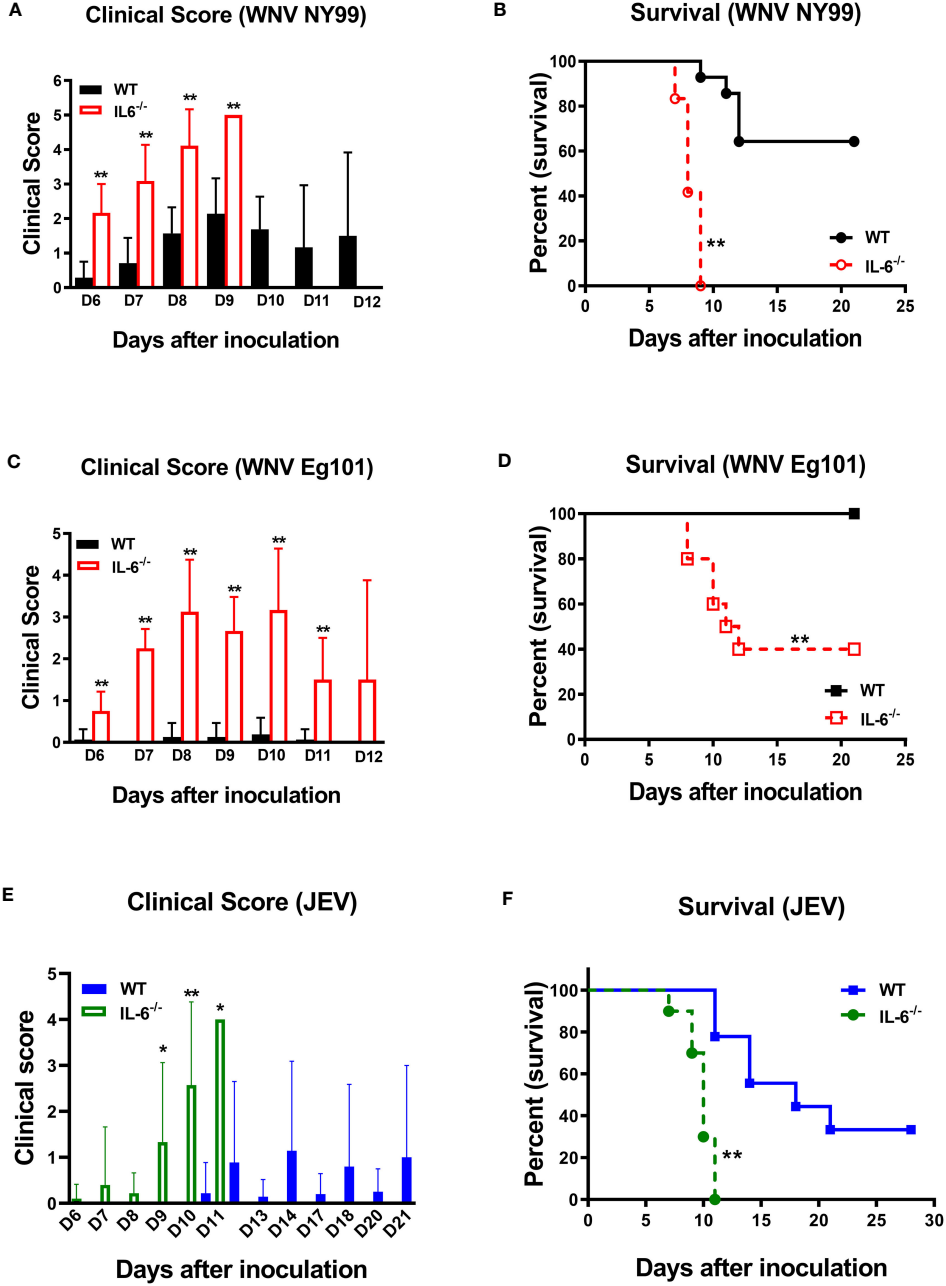
Clinical symptoms and survival of WT and IL-6^−/−^ mice following WNV or JEV infection. WT and IL-6^−/−^ mice were inoculated via footpads with **(A, B)** WNV NY99 (100 PFU), **(C, D)** WNV Eg101 (1,000 PFU), or **(E, F)** JEV Nakayama (1,000 PFU) strain. **(A, C, E)** Animals were checked daily for clinical signs. The clinical scores are as follows: 1 = ruffled fur/hunched posture; 2 = lethargy; 3 = abnormal walking; 4 = tremors/paralysis/moribund (euthanized); and 5 = dead. Error bars indicate SD. **(B, D, F)** The difference between WT and IL-6^−/−^ mice survival was determined. *n* = 12-20 mice per group, *p < 0.05, **p < 0.01.

Next, to determine the role of IL-6 in WNV pathogenesis *in vivo*, we evaluated the morbidity and mortality of C57BL/6 (wild type) and IL-6^−/−^ mice after WNV infection. Eight-week-old mice were subcutaneously inoculated with the lethal WNV strain NY99 (100 PFU) or the non-lethal WNV strain Eg101 (1,000 PFU) and monitored daily till day 21. Our records of clinical scores showed that all IL-6^−/−^ mice manifested severe neurological symptoms after inoculation with WNV NY99 or WNV Eg101 (**Figures 3A, C**). These symptoms included ruffled fur, hunched posture, ataxic gait, tremors, paralysis, and death. However, most WT mice infected with WNV NY99 had moderate clinical symptoms, while the WT mice infected with WNV Eg101 did not display any significant clinical symptoms (**Figures 3A, C**).

The survival curve demonstrated that 100 PFU of WNV NY99 led to 40% mortality in WT mice, whereas it caused 100% mortality in IL-6^−/−^ mice (**Figure 3B**). The median survival time was also shorter for infected IL-6^−/−^ mice than WT mice. As expected, there were no fatalities among WT mice infected with 1,000 PFU of WNV Eg101. However, infection of IL-6^−/−^ mice with WNV Eg101 resulted in 60% mortality (**Figure 3D**). These findings suggest that IL-6 is necessary for the survival of mice following WNV infection.

Subsequently, we inoculated eight-week-old WT and IL-6^−/−^ mice subcutaneously with the JEV Nakayama strain (1,000 PFU) to assess the role of IL-6 in JEV pathogenesis *in vivo*. The clinical score data revealed that all IL-6^−/−^ mice and some WT mice developed severe neurological symptoms after infection (**Figure 3E**). Based on the survival curve, the infectious dose of 1,000 PFU resulted in 66% mortality in WT mice, whereas it caused 100% mortality in IL-6^−/−^ mice (**Figure 3F**). Like WNV infection, the median survival time was shorter for JEV-infected IL-6^−/−^ mice than WT mice. The high morbidity and mortality observed in JEV-infected IL-6^−/−^ mice suggest that IL-6 has a protective role in mice after peripheral JEV infection.

### IL-6 is required for the control of WNV and JEV load in the periphery and brain of mice

**Figure 4:**
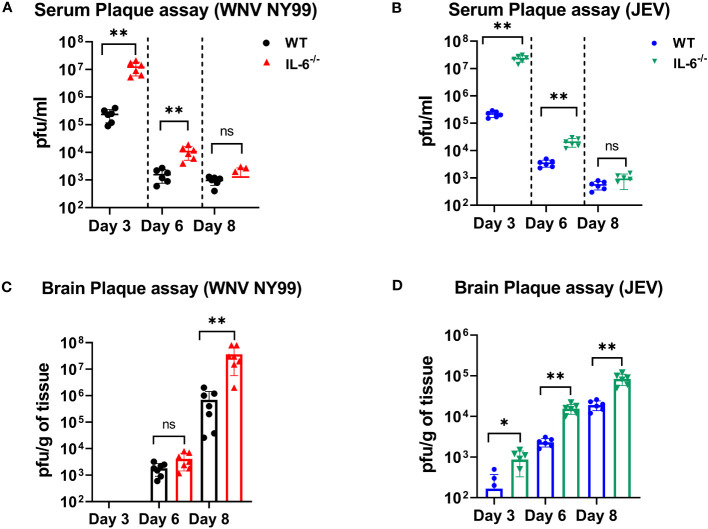
Evaluation of virus titers in WT and IL-6^−/−^ mice. Virus titers were determined in the serum on days 3, 6, and 8 after infection with **(A)** WNV NY99 or **(B)** JEV Nakayama by performing plaque assay, and results are expressed as PFU/mL ± SD **(C, D)** Virus titers in brain homogenates were assessed on days 3, 6, and 8 following WNV NY99 or JEV Nakayama infection and expressed as PFU/g of tissue ± SD. Each data point corresponds to an individual mouse (*n* = 6–7 mice per group). *p < 0.05, **p < 0.01.

We measured the viral titers in the serum and brains of WT and IL-6^−/−^ mice at different time points following subcutaneous infection with WNV NY99 or JEV Nakayama strain by performing plaque assays. We detected significantly higher WNV or JEV titers in the serum of IL-6^−/−^ mice compared to WT mice on day 3. The viral load decreased from day 3 to 6 in both groups; however, it remained significantly higher in IL-6^−/−^ mice compared to the WT mice. We observed low viremia on day 8; however, the difference between IL-6^−/−^ mice and WT mice was not significant (**Figures 4A, B**).

Next, we examined the viral load in the brains harvested on days 3, 6, and 8 after WNV or JEV infection using plaque assay. WNV was not found in brains on day 3. WNV was detected on day 6 in both sets of mice, with no significant difference between the groups. However, WNV loads were significantly higher in the brains of IL-6^−/−^ mice than in WT mice on day 8 (**Figure 4C**). Furthermore, IL-6^−/−^ mice exhibited significantly higher JEV loads in brains than WT mice at all time points (**Figure 4D**). These data suggest that IL-6 controls WNV and JEV replication in both the periphery and the brains of mice.

### Innate immune responses in WT and IL-6^−/−^ mice after WNV infection

**Figure 5:**
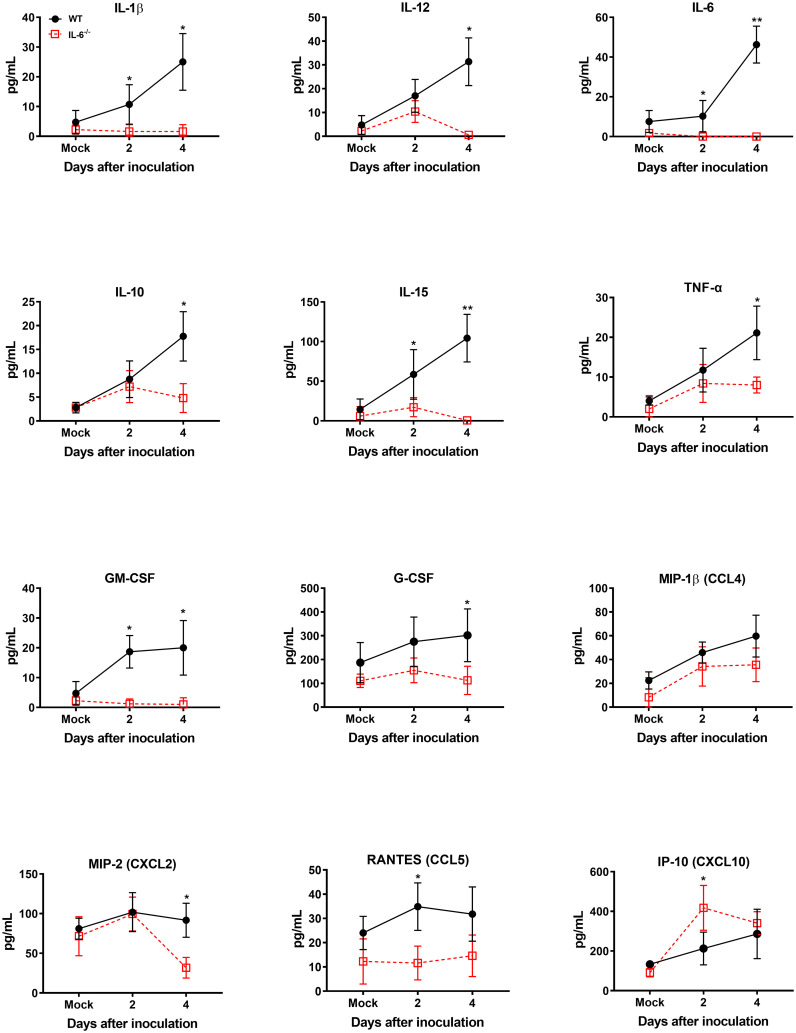
Analysis of cytokine and chemokine protein levels in the serum of WT and IL-6^−/−^ mice after infection with WNV NY99. Cytokine and chemokine levels were assessed in the serum of WNV NY99-infected WT and IL-6^−/−^ mice on days 2 and 4 following infection by performing a multiplex immunoassay. The data are expressed as the mean concentration (pg/mL) ± SD (*n* = 6–7 mice per group). *p < 0.05, **p < 0.01.

It is known that WNV infection causes significant upregulation of numerous cytokines and chemokines (Garcia-Tapia et al., 2007). WNV-induced proinflammatory mediators protect hosts from lethal WNV infection (Suthar et al., 2013). However, sustained activation and dysregulation of these host factors can also result in fatal WNV disease (Samuel and Diamond, 2006). To evaluate the role of IL-6 in modulating key cytokines and chemokines in WNV-infected mice, first, we assessed the levels of these proteins in the serum of WT and IL-6^−/−^ mice on days 2 and 4 using a multiplex immunoassay. Interestingly, the protein levels of IL-1β, IL-10, IL-12, IL-15, TNF-α, GM-CSF, G-CSF, MIP-2 (CXCL2), and RANTES (CCL5) were significantly lower in IL-6^−/−^ mice than WT mice (**Figure 5**). There are evidence that IL-6 induces several cytokine and chemokine pathways in the periphery (Caiello et al., 2014; Tanaka et al., 2014). Interestingly, the IP-10 (CXCL10) protein level was significantly elevated in IL-6^−/−^ mice than in WT mice.

**Figure 6:**
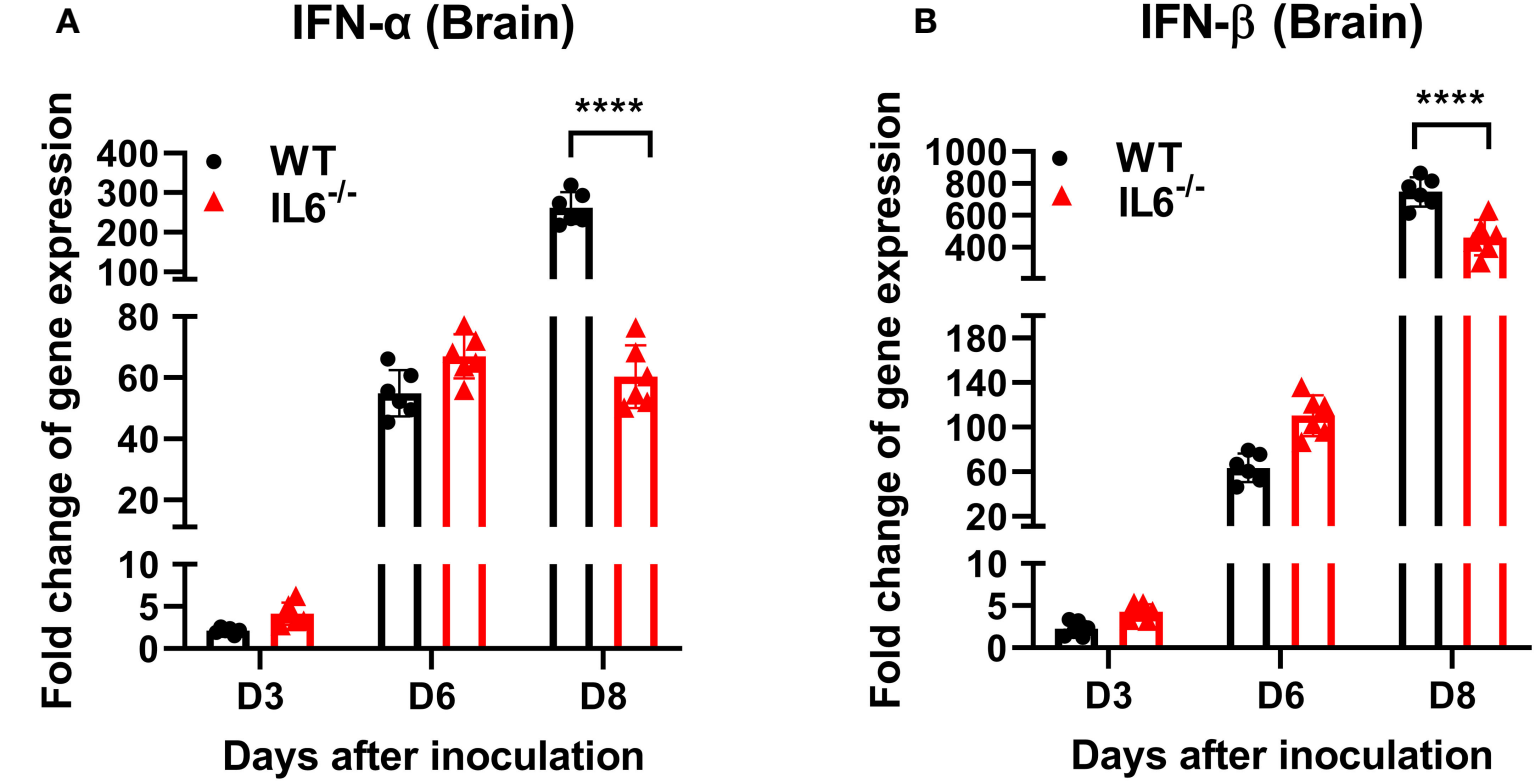
Type I interferon mRNA expression in the brains of WT and IL-6^−/−^ mice after WNV NY99 infection. Total RNA was extracted, and cDNA templates were generated from brain homogenates of WT and IL-6^−/−^ mice at days 3, 6, and 8 after infection. Quantification of **(A)** IFN-α and **(B)** IFN-β gene expressions were conducted using qRT-PCR. Expression levels were normalized to the housekeeping gene, GAPDH, and fold changes in infected cells relative to mock-infected controls were determined. Data is presented as mean ± SD from three independent experiments conducted in duplicate (n = 6 mice per group). ****p < 0.0001.

Type I interferon (IFN-I) response is essential for protecting the brain from neurotropic flaviviruses (Samuel and Diamond, 2005; Weber et al., 2014; Lindqvist et al., 2016; Fares et al., 2020). We next investigated whether IL-6 deficiency affects IFN-I activity in mice following WNV NY99 infection. We performed qRT-PCR to measure the expression levels of IFN-α and IFN-β in brain homogenates collected from WT and IL-6^−/−^ mice at 3-, 6-, and 8 days after infection. Our data demonstrated a significant reduction in the expression of IFN-α (**Figure 6A**) and IFN-β (**Figure 6B**) genes in IL-6^−/−^ brains compared to WT brains at day 8 after infection.

**Figure 7:**
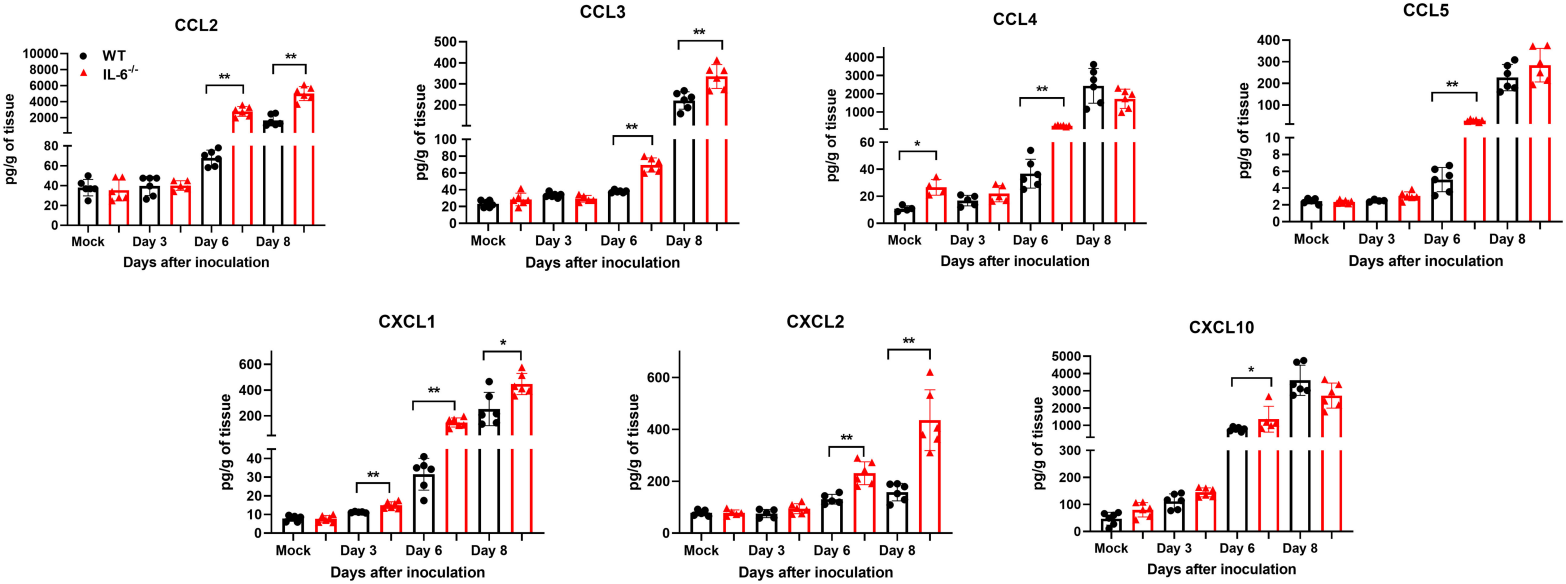
Analysis of chemokine concentrations in murine brains following infection with WNV NY99. Multiplex immunoassay was conducted to measure the protein levels of chemokines in the brains of WT and IL-6^−/−^ mice on days 3, 6, and 8 after WNV NY99 infection. The results were presented as the mean concentration (pg/g) of tissue ± SD (*n* = 6–7 mice per group). *p < 0.05, **p < 0.01.

**Figure 8:**
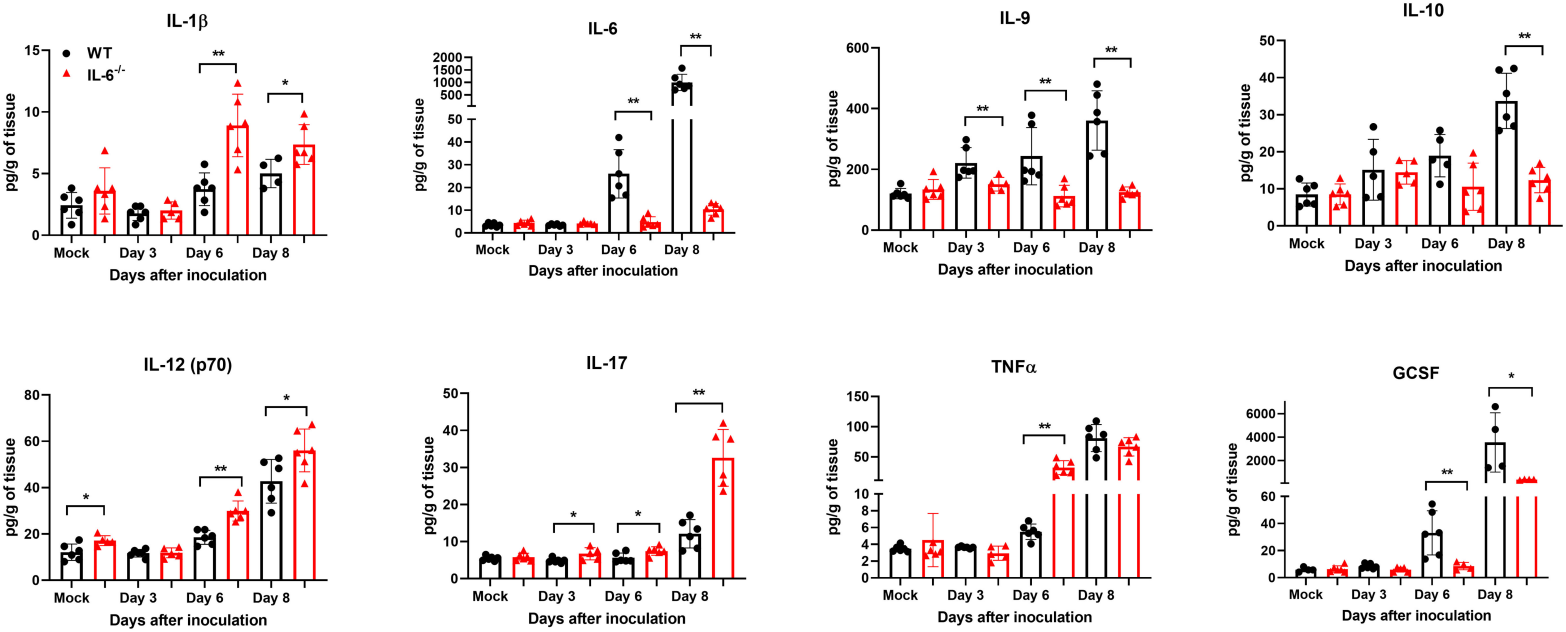
Analysis of cytokine concentrations in murine brains following infection with WNV NY99. Multiplex immunoassay was conducted to measure the protein levels of cytokines in the brains of WT and IL-6^−/−^ mice on days 3, 6, and 8 after WNV NY99 infection. The results were presented as the mean concentration (pg/g) of tissue ± SD (*n* = 6–7 mice per group). *p < 0.05, **p < 0.01.

Next, we measured the protein levels of essential cytokines and chemokines in the brain homogenates of WT and IL-6^−/−^ mice on days 3, 6, and 8 upon WNV infection (**Figures 7**, **8**). The multiplex immunoassay revealed that the protein concentrations of chemokines, CCL2, CCL3, and CXCL2, were significantly higher in the brains of IL-6^−/−^ mice than WT mice on days 6 and 8 (**Figure 7**). For CCL4, CCL5, and CXCL10, the brains of IL-6^−/−^ mice had higher protein levels on day 6. Interestingly, the CXCL1 protein, known for recruiting and activating neutrophils at the infected tissue site (Sawant et al., 2016), was substantially elevated in IL-6^−/−^ mice at all time points. Similarly, protein concentrations of IL-1β and IL-12 (p70) were significantly higher in IL-6^−/−^ mice brains on days 6 and 8 (**Figure 8**). Notably, TNF-α protein level was significantly elevated in the brains of IL-6^−/−^ mice than WT mice only at day 6. However, the IL-17 protein, which activates local chemokine production (Zenobia and Hajishengallis, 2015), was significantly increased in IL-6^−/−^ mice brains compared to WT mice brains at all time points. As expected, IL-6 protein concentration was significantly reduced in IL-6^−/−^ mice brains. Interestingly, protein levels of IL-9, GCSF, and IL-10 were significantly decreased in the brains of IL-6^−/−^ mice. These three cytokines have anti-inflammatory functions (Iyer and Cheng, 2012; Karagiannis and Wilhelm, 2017) and prevent excess infiltration of phagocytic cells to the infected tissue sites (Fang et al., 2018). Our findings in this experiment highlight the role of IL-6 in modulating the immune response to flavivirus infection in mice by differential regulation of type I interferon, cytokines, and chemokines in the periphery and brain.

## Discussion

IL-6 is a cytokine exerting pleiotropic effects. In the CNS, IL-6 is a neurotropic factor produced by neurons and glial cells, promoting neuronal survival, nerve regeneration, glial cell activation, and astrocyte proliferation while suppressing demyelination (Selmaj et al., 1990; Hama et al., 1991; Merrill, 1992; Campbell et al., 1993; Chiang et al., 1994; Rodriguez et al., 1994; Steffensen et al., 1994; Fattori et al., 1995; Klein et al., 1997; März et al., 1997; Loddick et al., 1998). Several studies have shown that impaired IL-6 function enhances susceptibility to infection with various pathogens. For instance, IL-6 deficiency increased mortality in mice after infection with HSV (LeBlanc et al., 1999; Murphy et al., 2008) and influenza virus (Lauder et al., 2013; Yang et al., 2017). Conversely, when WT mice were infected with a genetically modified rabies virus carrying the IL-6 gene, they exhibited greater resistance to viral infection than the parental virus (Luo et al., 2018). Additionally, infections with bacteria, such as *Streptococcus pyogenes* (Diao and Kohanawa, 2005), *Streptococcus pneumoniae* (van der Poll et al., 1997), *M. tuberculosis* (Ladel et al., 1997), and *T. cruzi* (Sanmarco et al., 2017), were more severe in IL-6^−/−^ mice at similar inoculation titers than WT mice. However, the effects of IL-6 on flavivirus infection have not been analyzed.

WNV and JEV preferentially infect myeloid cells (lymphoid dendritic cells and macrophages) from peripheral tissues and neuronal cells *in vivo* (Shrestha et al., 2003; Wang and Deubel, 2011; Zimmerman et al., 2019). However, the specific impact of IL-6 on flavivirus-infected myeloid and neuronal cells has never been studied before. Our study using human neuronal cells demonstrated significantly increased virus titers in WNV or JEV-infected cells when IL-6 was neutralized, indicating a restrictive function of IL-6 in flavivirus replication. To aid our *in vitro* human data, we infected primary mouse cells derived from the peripheral tissues and brain with a lethal or non-lethal WNV strain or JEV Nakayama strain. Similarly, we found significantly higher viral yields in the cells derived from IL-6^−/−^ mice compared to cells from WT mice. These observations suggest that IL-6 limits flavivirus production in both peripheral and CNS cells. Our results align with previous investigations that have shown adverse effects of IL-6 neutralization on virus clearance in cell-based studies involving hepatitis B virus (Kuo et al., 2009) and *Listeria monocytogenes* infection (Lücke et al., 2018). It has also been demonstrated that cells lacking similar cytokines, such as TNF-α and IL-1β, are more susceptible to WNV infection (Cheng et al., 2004; Ramos et al., 2012).

Mice infected with flavivirus exhibit severe neurological complications that closely resemble human diseases, rendering mice an excellent model for unraveling the molecular mechanisms of WNV or JEV infection (Graham et al., 2017; Frank et al., 2023). Therefore, we used C57BL/6J (WT) and IL-6^−/−^ mice for our experiments. Our *in vivo* studies revealed that the peripheral inoculation of the lethal NY99 strain of WNV or JEV Nakayama strain caused 100% mortality in IL-6^−/−^ mice, highlighting a significant difference in survival rates compared to WT mice. Interestingly, these findings contrast with a previous work, which implied that WNV infection does not cause any significant difference in survival between WT and IL-6^−/−^ mice (Wang et al., 2004). To further dissect the pathogenesis of WNV in IL-6^−/−^ mice, we subcutaneously inoculated WT and IL-6^−/−^ mice with WNV Eg101, a non-lethal strain (Shirato et al., 2004; Kumar et al., 2014). Consistent with our WNV NY99 data, IL-6^−/−^ mice exhibited a substantial difference from WT mice in mortality rates (60% vs 0%). Collectively, our survival data indicated a protective role for IL-6 against the pathogenic effects of WNV or JEV infection in mice. Subsequent plaque assay analyses showed that the viral titers were significantly higher in the serum and brains of WNV or JEV-infected IL-6^−/−^ mice than those of WT mice. These results align with prior studies that reported increased virus titers in IL-6^−/−^ mice after infection with influenza virus (Lauder et al., 2013; Yang et al., 2017), HSV-1 (LeBlanc et al., 1999), vaccinia virus (Kopf et al., 1994), *E. coli* (Dalrymple et al., 1996), *S. pneumoniae* (van der Poll et al., 1997; Schmit et al., 2020), and *Candida albicans* (Van Enckevort et al., 1999). Moreover, it is also known that animals lacking essential cytokine functions, such as TNF-R1^−/−^ and IL-1r^−/−^ mice, exhibit significantly increased mortality and elevated WNV titers in the CNS compared to WT mice (Shrestha et al., 2008; Ramos et al., 2012).

IFN-I activation is a vital defense against viral infections (Müller et al., 1994). Mice lacking the IFN-α/β receptor (IFN-αβR^−/−^) exhibit widespread tissue invasion, uncontrolled viral replication, and increased mortality compared to WT mice following WNV infection (Samuel and Diamond, 2005). Experiments with IFN-β receptor-deficient mice (IFN-β^−/−^) demonstrated that IFN-β controls WNV pathogenesis in mice by limiting infection in a cell and tissue-specific manner (Lazear et al., 2011). Furthermore, studies conducted *in vitro* have shown that treating primary neurons with IFN-β before and after WNV infection boost neuronal survival (Samuel and Diamond, 2005). Moreover, impaired IFN-I responses rendered neurons and astrocytes more susceptible to TBEV (Tick-borne encephalitis virus) infection (Lindqvist et al., 2016; Fares et al., 2020), and increase the spread of Langat virus (LGTV) within the CNS (Weber et al., 2014). It is established that basal IFN-I activity facilitates IL-6 expression (Ito et al., 1996; FUJISAWA et al., 1997; Murray et al., 2015; Zimmermann et al., 2016). Evidence shows that cells lacking the IFNAR1 receptor (IFNAR1^−/−^) provide fewer docking sites for STAT1 and STAT3, thus impairing the STAT1/3 pathway and, consequently, affecting IL-6 signaling (Mitani et al., 2001). However, the interplay between IFN-I and IL-6 during flavivirus infection is yet to be explored. Our *in vivo* data demonstrated that expression levels of IFN-α and IFN-β in the brain homogenates were initially increased on days 3 and 6 but significantly decreased on day 8 in IL-6^−/−^ mice compared to the WT mice. These findings indicate that the lack of IL-6 gene restricted the antiviral response induced by type I IFN in mouse brains after flavivirus infection.

IL-6 initiates signaling cascade events mainly via the JAK/STAT3 activation pathway (Wang et al., 2013). These phenomena stimulate the transcription of diverse downstream genes, including cytokines, chemokines, receptors, adaptor proteins, and protein kinases (Velazquez-Salinas et al., 2019). To evaluate the role of IL-6 in modulating the immune response after flavivirus infection, we measured the levels of key cytokines after IL-6 neutralization in human neuronal cells. Interestingly, despite having higher viral loads, we observed a significant depletion in the expression of cytokines, TNF-α and IL-1β, in the infected cells. Next, we measured the protein levels of cytokines and chemokines in WT and IL-6^−/−^ mice using multiplex immunoassay. Similarly, our results showed a significantly lower production of key cytokines and chemokines, such as IL-1β, IL-10, IL-12, IL-15, TNF-α, GM-CSF, G-CSF, MIP-2 (CXCL2), and RANTES (CCL5), in the serum of IL-6^−/−^ mice, even though the serum had high virus titers. It may be due to the impaired IL-6-mediated activation of cytokine/chemokine signaling pathways in the periphery (Caiello et al., 2014; Tanaka et al., 2014). IL-6 is a master regulator and modulates the secretion of other cytokines. Therefore, the absence of IL-6 might have suppressed cytokine and chemokine induction in the periphery of IL-6^−/−^ mice. Interestingly, we found that the protein levels of some cytokines (IL-1β, IL-12, and IL-17) and chemokines (CCL2, CCL3, CXCL1, and CXCL2) were significantly elevated in the brains of IL-6^−/−^ mice than in WT mice. Conversely, IL-9, IL-10, and GCSF protein levels were significantly decreased in the brains of IL-6^−/−^ mice, which have anti-inflammatory roles (Hartung, 1998; Iyer and Cheng, 2012; Karagiannis and Wilhelm, 2017). The elevated production of some cytokines and chemokines in the brains of IL-6^−/−^ mice could be attributed to the increased immune cell infiltration and the diminished ability to control virus replication due to decreased type I IFN levels. Elevated levels of these inflammatory molecules in the brain correlate with the increased mortality observed in the IL-6^−/−^ mice. Overall, our data indicate the pivotal role of IL-6 in modulating the immune response to flavivirus infection in both human neuronal cells and mice. Further mechanistic studies are needed to unravel the complex interactions between IL-6 and type I IFN and key cytokines in the periphery and CNS of flavivirus-infected mice.

In conclusion, our findings offer novel insights into the role of IL-6 during flavivirus replication and dissemination. Our report shows, for the first time, that lack of IL-6 increases the severity of WNV or JEV infection both *in vitro* and *in vivo*. Future mechanistic studies on the function of IL-6 during neurotropic flavivirus infection will significantly impact the development of much-needed therapeutic interventions to improve disease outcomes.

## Data availability statement

The original contributions presented in the study are included in the article/supplementary material. Further inquiries can be directed to the corresponding author.

## Ethics statement

Ethical approval was not required for the studies on humans in accordance with the local legislation and institutional requirements because only commercially available established cell lines were used. The animal study was approved by Georgia State University IACUC. The study was conducted in accordance with the local legislation and institutional requirements.

## Author contributions

TA: Data curation, Formal Analysis, Investigation, Methodology, Validation, Visualization, Writing – original draft, Writing – review and editing. KA: Conceptualization, Data curation, Formal Analysis, Investigation, Methodology, Project administration, Software, Supervision, Validation, Visualization, Writing – original draft, Writing – review and editing. JN: Data curation, Formal Analysis, Investigation, Methodology, Software, Validation, Writing – review and editing. HP: Data curation, Formal Analysis, Investigation, Methodology, Software, Writing – review and editing. AE: Formal Analysis, Investigation, Methodology, Software, Writing – review and editing. MK: Conceptualization, Data curation, Formal Analysis, Funding acquisition, Investigation, Methodology, Project administration, Resources, Supervision, Validation, Visualization, Writing – original draft, Writing – review and editing.
